# Fenaminosulf Promotes Growth and Gall Formation in *Zizania latifolia* Through Modulation of Physiological and Molecular Pathways

**DOI:** 10.3390/plants14111628

**Published:** 2025-05-27

**Authors:** Chaohong Ding, Ruifang Ma, Liqiu Wang, Xinyan Lan, Limin Chen, Jinxing Zhu, Lailiang Wang

**Affiliations:** 1 Lishui Institute of Agriculture and Forestry Sciences, Lishui 323000, China; lsdch_74@163.com (C.D.); mareifang@126.com (R.M.); clmit@zju.edu.cn (L.C.); 2 College of Ecology, Lishui University, Lishui 323000, China; wanglq0909@163.com (L.W.); lanxya4086@163.com (X.L.); 3 Suichang Soil Fertilizer and Plant Protection and Energy Sources Station, Lishui 323000, China

**Keywords:** *Zizania latifolia*, fenaminosulf, transcriptomics, gall formation, plant growth regulation, hormone signaling

## Abstract

*Zizania latifolia* (Jiaobai) is an economically important aquatic crop characterized by unique gall formation through interaction with the smut fungus *Ustilago esculenta*. Understanding factors influencing this interaction is crucial for cultivation. This study investigates the non-target effects of the fungicide Fenaminosulf (FM) on *Z. latifolia*’s growth, physiology, and underlying molecular pathways. We demonstrate that FM exerts striking concentration-dependent effects, revealing its potential as a modulator of plant development and symbiosis. Physiological measurements showed that a moderate FM concentration (1.25 g/L) promoted key vegetative growth parameters, including plant height and leaf length, while maintaining chlorophyll content, suggesting a potential bio-stimulant effect. In contrast, higher FM concentrations (2.5 g/L and 5 g/L) inhibited vegetative growth but significantly enhanced gall formation, particularly at 2.5 g/L, indicating that FM can redirect plant resources or alter susceptibility to favor the fungal interaction under specific conditions. Transcriptomic analysis provided mechanistic insights, revealing extensive gene expression reprogramming, especially under high FM treatment (5 g/L). Key pathways related to plant-pathogen interaction, phenylpropanoid biosynthesis, and hormone signal transduction were significantly modulated. Notably, FM treatment suppressed key immune-related genes, including *Xa21* and *PBL19*, potentially reducing plant resistance and facilitating gall formation. Hormone signaling analysis revealed inhibition of auxin, cytokinin, brassinosteroid, and jasmonic acid metabolism, indicating a comprehensive molecular recalibration of plant developmental processes. The study provides novel insights into the molecular mechanisms by which FM influences *Z. latifolia* growth and gall formation. The concentration-dependent effects of FM suggest its potential as a strategic tool for agricultural management, offering a nuanced approach to crop development. These findings contribute to understanding plant-chemical interactions and provide valuable directions for optimizing *Z. latifolia* cultivation strategies.

## 1. Introduction

*Zizania latifolia* (Griseb.), commonly known as water bamboo or Jiaobai [1], is an important aquatic plant cultivated for over 2000 years in China and widely consumed across East and Southeast Asia [1,2]. Unlike typical cereal crops, *Z. latifolia* undergoes a unique morphological transformation when parasitized by the smut fungus *Ustilago esculenta* [3]. Instead of producing flowers and seeds, the plant develops an enlarged edible pseudostem, making it a valuable vegetable crop [4,5]. The interaction between *U. esculenta* and *Z. latifolia* is critical to its commercial production, as fungal colonization induces significant changes in plant growth and development, leading to gall formation [5]. Despite its economic and agricultural importance, the underlying mechanisms regulating this transformation remain largely unexplored.

Recent studies have highlighted the complex interplay between chemical agents and plant physiological processes, particularly in the context of growth regulation and stress responses [6]. Fungicides such as sodium disulfinate also known as fenaminosulf (FM), traditionally used to combat fungal pathogens [7], have been shown to exert additional effects on plant development, including accelerated growth and gall formation in *Z. latifolia* [8,9]. These effects are thought to be mediated through the modulation of plant hormone signaling pathways, metabolic processes, and defense mechanisms, although the precise molecular mechanisms remain poorly understood [8]. Plant hormones, including brassinosteroids, auxins, and cytokinins, play pivotal roles in regulating growth, development, and stress responses [10,11,12,13,14], suggesting a potential link between FM treatment and hormonal crosstalk in *Z. latifolia* [15]. Furthermore, the antagonistic and synergistic interactions between these hormones, as well as their influence on plant-pathogen interactions, provide a plausible framework for understanding how FM may induce physiological changes [12,14,16].

Fungicides are believed to affect several key biological processes, including plant hormone metabolism, phenylpropanoid biosynthesis, and plant defense signaling [17]. The phenylpropanoid pathway, in particular, plays a crucial role in plant immune responses, producing compounds that regulate resistance against pathogens [5,14]. Research has shown that external chemical treatments can either enhance or suppress plant defense mechanisms [17,18], thereby influencing fungal colonization and gall formation. Given the delicate balance between fungal infection and plant growth in Jiaobai production, understanding the role of sodium disulfinate in these processes is essential for optimizing cultivation practices. Furthermore, KEGG pathway enrichment analyses have revealed that genes related to hormone signal transduction and metabolic regulation are differentially expressed under FM treatment, indicating its potential impact on key regulatory networks [15].

Despite its widespread use in agricultural production, there is limited research on the mechanistic effects of sodium disulfinate in *Z. latifolia*. While it has been shown to promote Jiaobai growth and advance harvest time, its mode of action at the molecular and physiological levels remains largely unknown. This study aims to investigate the effects of sodium disulfinate treatment on the growth, hormonal regulation, and transcriptomic changes in *Z. latifolia*. By examining its impact on key metabolic pathways and plant defense responses, this research seeks to provide new insights into the mechanisms by which chemical treatments influence Jiaobai development. A deeper understanding of these processes will contribute to improved cultivation strategies and potentially lead to the development of novel agricultural applications for controlling plant growth and disease resistance.

## 2. Results

### 2.1. Effects of Fenaminosulf Treatment on the Growth of Zizania latifolia

**Figure 1:**
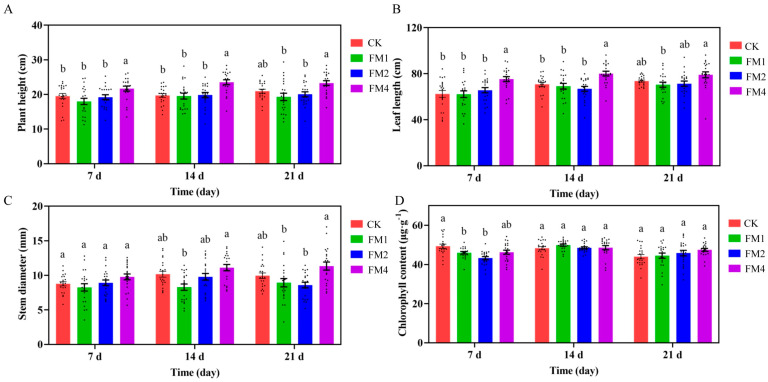
Effects of Fenaminosulf (FM) treatment on the growth parameters of *Z.latifolia*. (**A**) Plant height measured at 7, 14, and 21 days after foliar application of different FM concentrations (FM1: 5 g/L, FM2: 2.5 g/L, FM4: 1.25 g/L) compared to the control (CK). (**B**) Leaf length response to different FM concentrations over time. FM4 significantly promoted leaf elongation, whereas higher concentrations did not show a consistent effect. (**C**) Stem thickness recorded at 7-, 14-, and 21-days post-treatment. (**D**) Chlorophyll content in *Z. latifolia* leaves after FM treatment. Different letters indicate statistically significant differences between treatments at each time point (*p* < 0.05, one-way ANOVA followed by Tukey’s HSD test). Data are presented as means ± SE (*n* = 3 biological replicates per treatment).

The application of different concentrations of Fenaminosulf (FM) had a significant impact on the growth parameters of *Z. latifolia*, including plant height, leaf length, stem diameter, and chlorophyll content (Figure 1). Seven days post-treatment, plants treated with 1.25 g/L (FM4) showed a significant increase in height compared to the control group (CK), suggesting that a moderate concentration of FM can enhance growth. In contrast, plants exposed to higher FM concentrations (5 g/L FM1 and 2.5 g/L FM2) exhibited non-significant reduction in height.

Leaf length followed a similar trend, with 1.25 g/L FM4 significantly promoting leaf elongation, whereas higher concentrations had little to no effect. Stem thickness showed no significant differences when compared to the control. However, significant differences emerged when comparing FM4 to FM1 at 14- and 21-days post-treatment, indicating that excessive FM application may have an inhibitory effect on stem growth.

Chlorophyll content analysis revealed that high concentrations of FM (FM1 and FM2) significantly reduced chlorophyll levels and photosynthetic capacity, whereas FM4 had no negative effect and instead exhibited an increasing trend over time. These findings suggest that moderate concentrations of FM promote the growth and development of *Z. latifolia*, whereas excessive concentrations have inhibitory effects.

### 2.2. Effects of Fenaminosulf on Gall Formation in Z. latifolia

**Figure 2:**
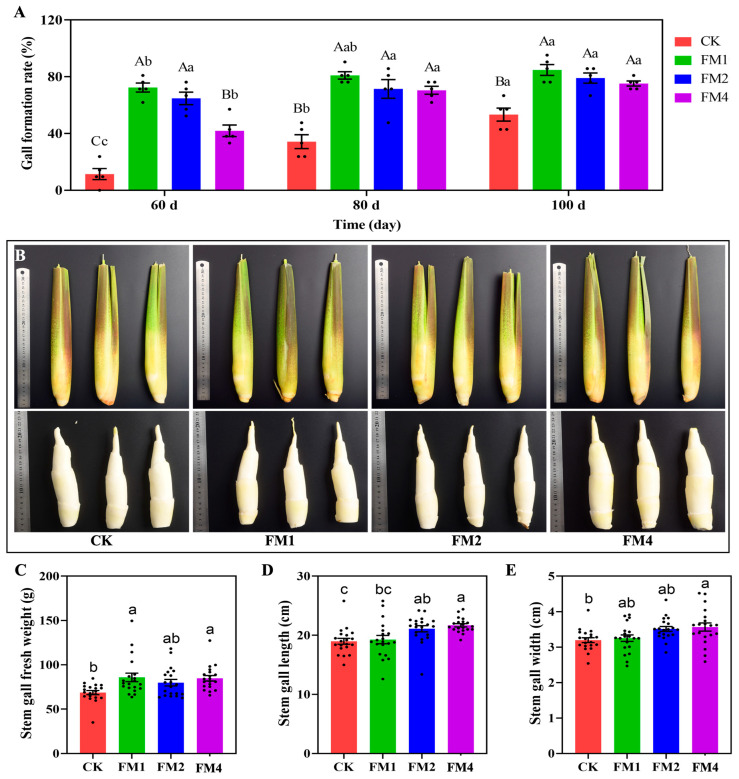
Effects of Fenaminosulf (FM) treatment on gall formation in *Z. latifolia*. (**A**) Gall formation rate (%) under control (CK), FM1, FM2, and FM4 treatments at 60, 80, and 100 days. Values are mean ± SE, with individual replicates shown as dots. Different uppercase letters indicate significant differences (*p* < 0.05) across time points for the same treatment, while different lowercase letters indicate significant differences (*p* < 0.05) among treatments at the same time point. (**B**) Representative images of shoots (top row) and the corresponding stem galls after leaf sheath removal (bottom row) for CK, FM1, FM2, and FM4 treatments. Rulers indicate scale in cm. (**C**) Stem gall fresh weight (g), (**D**) stem gall length (cm), and (**E**) stem gall width (cm) at 100 days for CK, FM1, FM2, and FM3 treatments. Values are mean ± SE, with individual replicates shown as dots. Different lowercase letters above bars denote significant differences (*p* < 0.05) among treatments.

One of the critical effects of FM treatment was its influence on gall formation in *Z. latifolia* (Figure 2A). After 60 days of treatment, the cumulative gall formation rate was significantly higher in plants treated with FM1 and FM2 compared to the control group (CK). Notably, the FM4 treatment, which showed positive effects on early growth, resulted in a lower gall formation rate compared to the moderate and high FM concentrations, suggesting that higher FM concentrations accelerate the induction of gall formation. Additionally, statistical analysis of the cumulative gall formation rate at different time points indicated that FM-treated groups showed a faster onset of gall development compared to the CK group, particularly in the FM1 and FM2 treatments.

Beyond the rate and onset of gall formation, the physical characteristics of the galls at 100 days were also significantly influenced by different FM treatments (Figure 2C–E). Specifically, stem gall fresh weight was significantly increased in the FM1 (average 85.83 g) and FM3 (average 84.79 g) treatments compared to CK (average 68.7 g), with FM2 (average 79.87 g) also showing a numerically higher average weight (Figure 2C). For stem gall length, both FM2 (average 21.10 cm) and FM3 (average 21.69 cm) treatments resulted in significantly longer galls than the CK group (average 18.98 cm), with FM3 showing the greatest length (Figure 2D). A similar trend was observed for stem gall width, where FM2 (average 3.51 cm) and FM3 (average 3.57 cm) treatments produced significantly wider galls compared to CK (average 3.20 cm), again with FM3 exhibiting the largest width (Figure 2E).

These findings suggest that Fenaminosulf plays a role in promoting gall formation, potentially through its interactions with hormonal pathways and plant-pathogen interactions.

### 2.3. Overview of Transcripotmic Data

The transcriptomic analysis of *Z. latifolia* involved sequencing nine libraries, each producing at least 6 Gb of clean data, with a Q30 percentage exceeding 96%, ensuring high base-calling accuracy (Table S1). Quality control was conducted using Fastp, which filtered out low-quality sequences, including adapter-containing reads, reads with more than 10% ambiguous bases (N), and those where over 50% of bases had a quality score of Q ≤ 20. The sequencing error rate was slightly elevated at the beginning of reads, likely due to the presence of random hexamer priming, but the GC content remained stable after the initial bias correction. The clean reads were aligned to the reference genome using HISAT2, achieving an average mapping efficiency of 89.6–90.1%, with uniquely mapped reads constituting approximately 86.9–87.6% across samples. The total number of reads obtained per sample ranged from 48,878,522 to 73,568,618, and after quality control, clean reads varied between 46,307,460 and 70,707,512 (Table S1). Gene expression levels were quantified using featureCounts and normalized using FPKM values. The expression distributions were assessed through density and violin plots, confirming similar expression levels across replicates.

**Figure 3:**
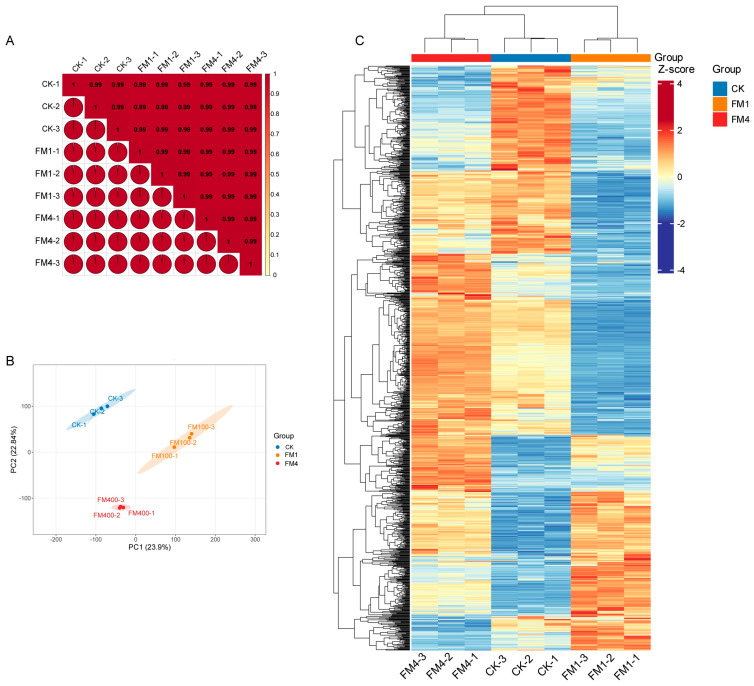
Overview of transcriptomic data quality and expression variation across treatments in *Z. latifolia*. (**A**) Pearson correlation heatmap of gene expression profiles among all biological replicates. Each cell represents the Pearson correlation coefficient (r) between two samples, with stronger positive correlations indicated by deeper red shades. (**B**) Principal Component Analysis (PCA) plot showing distinct clustering of samples based on treatment conditions. PC1 and PC2 explain 42.7% and 21.3% of the total variance, respectively, separating control (CK), FM1, and FM4 treatments. (**C**) Hierarchical clustering heatmap of DEGs across all samples, where rows represent genes and columns represent replicates. Expression levels are scaled and color-coded from blue (low) to red (high), illustrating clear treatment-specific expression patterns.

To evaluate the reliability of biological replicates, Pearson correlation coefficients were calculated, showing strong correlations between replicates within the same treatment group, with values 0.99, indicating high reproducibility (Figure 3A). Principal Component Analysis (PCA) revealed a clear separation of treatment groups, with PC1 accounting for 42.7% of the variance and PC2 explaining 21.3%, demonstrating distinct transcriptomic shifts in response to different treatments (Figure 3B). The clustering of replicates along these principal components further confirmed the robustness of the dataset and the consistency of transcriptomic changes induced by the treatments (Figure 3C).

### 2.4. Diffrential Expression Pattern FM-Treated Z. latifolia

**Figure 4:**
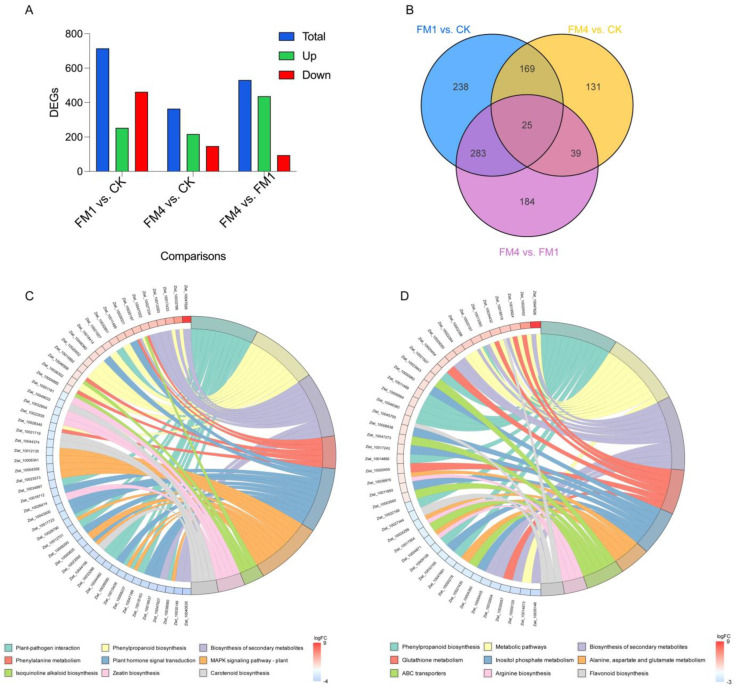
Differential dynamics of DEGs identified in FM-Treated *Z. latifolia* (**A**) Number of differentially expressed genes (up-regulated and down-regulated) in three pairwise comparisons (**B**) Venn diagram representing an overlap of DEGs in three pairwise comparisons (**C**) KEGG Pathways enrichment analysis for CK vs FM1 comparison (**D**) KEGG Pathways enrichment analysis for CK vs FM4 comparison.

Differential expression analysis was performed using DESeq2 for samples with biological replicates and edgeR for those without. A total of 715 differentially expressed genes (DEGs) were identified in FM1 vs. CK, with 253 upregulated and 462 downregulated genes (Figure 4A and Figure S1). In FM4 vs CK, 364 DEGs were detected, consisting of 217 upregulated and 147 downregulated genes. The FM4 vs FM1 comparison revealed 531 DEGs, with a striking pattern of 437 genes upregulated and 94 downregulated. The expression changes were visualized using MA plots, where log2 fold changes were plotted against gene expression levels, showing significant upregulation and downregulation trends. Volcano plots provided an overview of the distribution of DEGs, highlighting significantly altered genes with |log_2_Fold Change| ≥ 1 and FDR < 0.05. A clustering heatmap demonstrated distinct gene expression patterns, where samples grouped according to their treatment conditions, confirming consistent differential expression across biological replicates. K-means clustering of gene expression trends revealed distinct expression modules, capturing treatment-specific transcriptional responses.

A Venn diagram analysis illustrated the overlap of DEGs between different comparisons, showing that 25 genes were shared between three comparisons, while 238 DEGs were unique to FM1 vs CK, 131 DEGs were exclusive to FM4 vs CK, and 184 were unique to FM4 vs. FM1 (Figure 4B). The hierarchical clustering heatmap further confirmed the differentiation of expression profiles, with genes involved in stress response, metabolism, and transcriptional regulation exhibiting distinct treatment-specific expression patterns. These results highlight the significant transcriptomic alterations in *Z. latifolia* under different experimental conditions, providing valuable insights into the molecular mechanisms underlying its response to environmental stimuli.

### 2.5. KEGG Pathway Enrichment Analysis of Gene Expression in FM-Treated Z. latifolia

To further investigate the molecular mechanisms underlying FM-induced growth and gall formation, KEGG pathway enrichment analysis associated with differential expression patterns (Figure S1) was performed to examine differentially expressed genes (DEGs) in response to different FM concentrations (Figure S2).

**Figure 5:**
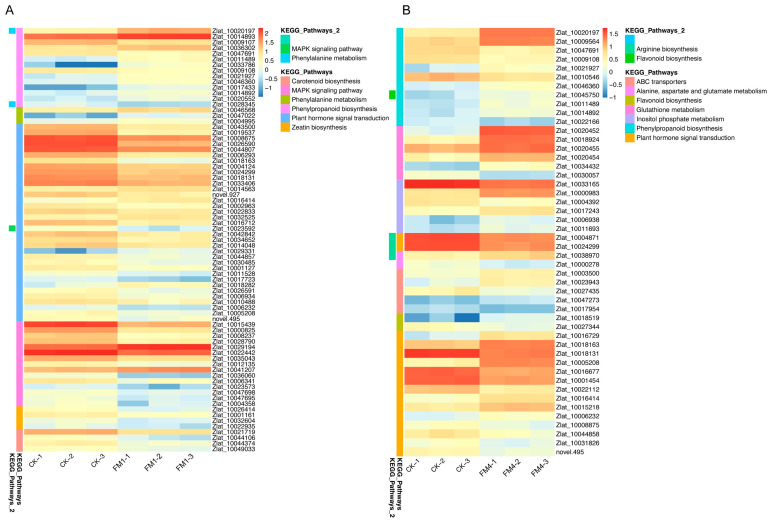
Expression dynamics of DEGs identified in FM-Treated *Z. latifolia*. Heatmaps illustrating the expression profiles of selected DEGs identified in the (**A**) Control (CK) vs. high concentration Fenaminosulf (FM1: 5 g/L) comparison, and (**B**) Control (CK) vs. moderate concentration Fenaminosulf (FM4: 1.25 g/L) comparison. Rows represent individual DEGs, labeled with their *Z. latifolia* gene identifiers. Columns represent the individual biological replicates for each treatment condition. Gene expression levels are represented by color intensity across all samples shown in each panel, where red indicates higher relative expression and blue indicates lower relative expression, according to the color key provided. Genes displayed are those belonging to significantly enriched KEGG pathways identified in the respective differential expression analyses (e.g., Phenylpropanoid biosynthesis, MAPK signaling, Hormone signal transduction, etc.), as indicated by the pathway annotations listed on the right side of each heatmap.

In plants treated with high concentrations of FM (FM1), DEGs were significantly enriched in pathways related to plant-pathogen interactions, phenylpropanoid biosynthesis, secondary metabolite biosynthesis, and plant hormone signal transduction (Figure 4C and Figure 5A, Table S2). Additionally, genes involved in the MAPK signaling pathway, carotenoid biosynthesis, and glutathione metabolism were significantly upregulated. In line with the KEGG enrichment analysis, detailed expression profiling of genes involved in phenylpropanoid biosynthesis and related pathways revealed strong transcriptional modulation under FM1 treatment. Several key genes in the phenylpropanoid pathway exhibited significant upregulation in FM1-treated plants compared to the control. For instance, *Zlat_10020197*, which participates in lignin precursor biosynthesis, showed a dramatic increase in expression from approximately 4 FPKM in CK to over 28 FPKM in FM1 samples, with a log_2_FoldChange of +2.83 and an adjusted *p*-value of 6.09 × 10^−57^. Similarly, *Zlat_10014893* and *Zlat_10036302* also demonstrated substantial upregulation, with log_2_FoldChanges of +1.04 and +1.31, respectively, reinforcing the FM1-induced activation of the phenylpropanoid biosynthetic pathway.

Interestingly, while a majority of genes within this pathway were induced, a subset showed downregulation. Notably, *Zlat_10009107* and *Zlat_10047691* were suppressed under FM1, with log_2_FoldChanges of −1.24 and −1.23, respectively. This indicates a nuanced, branch-specific regulation of the phenylpropanoid network, possibly reflecting a metabolic shift toward specific downstream compounds such as flavonoids, lignins, or coumarins. Additionally, several genes were mapped to both phenylpropanoid biosynthesis and phenylalanine metabolism, highlighting the interconnected nature of secondary metabolic routes. The coordinated upregulation of genes across these related pathways suggests that FM1 treatment enhances the biosynthesis of structural and defensive metabolites, likely contributing to the observed activation of plant-pathogen interaction and MAPK signaling pathways. These findings support the notion that FM1 triggers a broad-spectrum transcriptional reprogramming that primes *Z. latifolia* for enhanced stress resilience and metabolic adaptability.

Conversely, moderate FM concentrations (FM4) primarily affected genes associated with phenylpropanoid biosynthesis, flavonoid biosynthesis, ABC transporter activity, and photosynthesis (Figure 4D and Figure 5B, Table S3). Consistent with the trends observed under FM1 treatment, the FM4 concentration also led to significant transcriptional remodeling in *Z. latifolia*, particularly in pathways related to phenylpropanoid biosynthesis, secondary metabolite production, and defense responses. Within the phenylpropanoid biosynthesis pathway, multiple genes were markedly upregulated in FM4-treated plants. For example, *Zlat_10020197* exhibited a striking induction, with expression increasing from ~4 FPKM in control to over 22 FPKM in FM4 samples, corresponding to a log_2_FoldChange of +2.48 and an adjusted *p*-value of 1.45 × 10^−164^. Similarly, *Zlat_10009564* was significantly upregulated (log_2_FC = +1.51, FDR = 1.02 × 10^−41^), suggesting an intensified activation of lignin and flavonoid biosynthetic routes under FM4 stress.

Interestingly, this pathway also contained downregulated components. Notably, *Zlat_10047691* and *Zlat_10009108* both showed substantial suppression (log_2_FC = −1.19 and −1.16, respectively), indicating that while FM4 broadly stimulates phenylpropanoid biosynthesis, it may selectively repress certain branches, possibly as a mechanism to fine-tune resource allocation or flux toward specific compounds.

Furthermore, *Zlat_10021927*, which had minimal expression in the control group (~0.6 FPKM average), was significantly upregulated under FM4 (average ~2.3 FPKM), with a log_2_FoldChange of +1.75. These expression patterns, observed across several enzymes involved in lignin precursor formation, hydroxycinnamic acid metabolism, and related aromatic compound pathways, point toward a robust metabolic reprogramming driven by FM4 exposure. Altogether, these results suggest that FM4 induces a coordinated activation of phenylpropanoid metabolism alongside selective suppression of particular genes, possibly enhancing the biosynthesis of defense-related secondary metabolites such as lignin, coumarins, and flavonoids. This complex regulation supports a model in which FM4 strengthens structural integrity and stress tolerance in *Z. latifolia* through transcriptional reorganization of core metabolic pathways. 

Despite some differences between FM1 and FM4 treatments, both concentrations significantly affected plant defense-related pathways, particularly those associated with plant-pathogen interactions and phenylpropanoid biosynthesis (Figure S3 and Table S4). These results indicate that FM treatment influences multiple biological processes, including stress response pathways, hormonal signaling, and secondary metabolism, which may contribute to both the promotion of growth and the enhancement of gall formation in *Z. latifolia*.

### 2.6. Differential Expression of Genes Involved in Phenylpropanoid Biosynthesis Pathway

The phenylpropanoid biosynthesis pathway is a critical metabolic pathway involved in plant defense mechanisms and the production of secondary metabolites. Analysis of this pathway revealed that different FM concentrations led to significant changes in the expression of phenylpropanoid biosynthesis-related genes.

Interestingly, low concentrations of FM (FM4) were associated with the suppression of key genes in the phenylpropanoid pathway, indicating that reduced activity of this pathway may be linked to increased susceptibility to gall formation. On the other hand, higher FM concentrations (FM1 and FM2) triggered increased expression of phenylpropanoid biosynthesis genes (Figure 4C,D and Figure S4), possibly contributing to stress response activation and pathogen resistance mechanisms. These results suggest that FM plays a dual role in *Z. latifolia* by modulating phenylpropanoid metabolism, potentially reducing plant resistance while promoting conditions favorable for gall formation.

### 2.7. Differential Expression of Genes Involved in Plant–Pathogen Interactions

**Figure 6:**
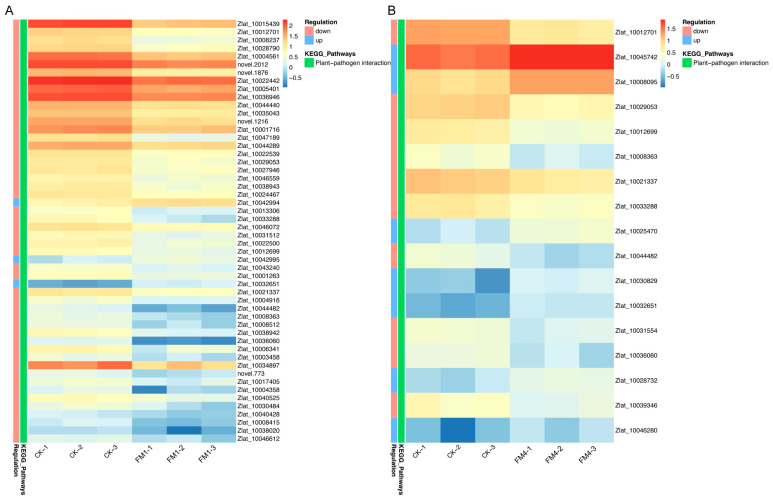
Modulation of the plant-pathogen interaction pathway by fenaminosulf treatment. Heatmaps showing the expression profiles of DEGs annotated to the Plant-Pathogen Interaction pathway (**A**) displays results for the CK vs. FM1 comparison, while panel (**B**) displays results for the CK vs. FM4 comparison. Rows represent individual pathway-associated DEGs, and columns represent biological replicates. Gene expression levels are represented by color intensity across all samples shown in each panel, where red indicates higher relative expression and blue indicates lower relative expression, according to the color key provided.

To investigate the role of FM treatments in modulating immune signaling, we focused on differentially expressed genes associated with the plant–pathogen interaction pathway in *Z. latifolia* under both FM1 and FM4 conditions (Figure 6). The data revealed a coordinated transcriptional modulation of key immune-related genes, including pattern recognition receptors (PRRs), calcium signaling components, defense regulators, and pathogenesis-related proteins.

In the FM1 vs CK comparison, genes involved in early pathogen recognition and downstream signaling were significantly downregulated (Figure 6A). Among them, the receptor-like kinase gene *Xa21* c*Zlat_10012701*), a critical component of PAMP-triggered immunity (PTI) [19], showed a strong reduction in expression under FM1 treatment, with a log_2_FC of −2.09 and an adjusted *p*-value of 4.11 × 10^−88^. This suggests that FM1 might suppress certain PRR-mediated responses, possibly as part of a broader transcriptional rebalancing. Additionally, several calcium-dependent protein kinases (CDPKs) and WRKY transcription factors displayed differential expression, indicating altered intracellular signaling dynamics under FM1.

A particularly notable gene was *PBL19* (*Zlat_10029053*), a probable serine/threonine kinase known to transduce pathogen signals downstream of PRRs [20], which showed consistent downregulation in both FM1 (log_2_FC = −1.58) and FM4 (log_2_FC = −1.43). Similarly, CML36 (*Zlat_10021337*), a calcium-binding protein linked to immune signal amplification, was also repressed in both treatments. Conversely, not all immune-related genes were downregulated. The wall-associated receptor kinase *WAK2* (*Zlat_10032651*) was significantly upregulated in both FM1 (log_2_FC = +1.49) and FM4 (log_2_FC = +2.27), possibly reflecting activation of mechanical stress-related defense pathways [21]. This suggests a potential shift from classical PRR-mediated immune pathways toward alternative perception or reinforcement strategies. In addition, the transcription factor *WRKY76* (*Zlat_10036060*), known for its dual role in regulating defense and abiotic stress responses [22], was sharply suppressed, particularly under FM4 (log_2_FC = −3.18), implying FM’s influence on transcriptional regulation of immune output.

Under FM4 treatment, *Xa21* was also significantly downregulated, though to a slightly lesser extent than in FM1 (Figure 6B). Its expression decreased from an average FPKM of ~19 in control to ~6 in FM4 samples, yielding a log_2_FC of −1.77 (FDR = 1.61 × 10^−85^). The consistent suppression of *Xa21* under both FM concentrations suggests a potential FM-mediated modulation of immune receptor signaling, possibly to avoid excessive immune activation or to prioritize other defense mechanisms. Interestingly, while *Xa21* was downregulated, several defense-associated transcription factors and pathogenesis-related (PR) genes were upregulated under FM4, indicating a shift in the immune response strategy.

Together, these findings reveal a complex and dose-dependent transcriptional reprogramming of the plant immune network under FM treatments. While key immune receptors like *Xa21* are suppressed, downstream defense-related pathways may be selectively activated, suggesting a reconfiguration of immune priorities in *Z. latifolia* under FM-induced stress (Figure S5A,B).

### 2.8. Effects of Fenaminosulf on Plant Hormone Signal Transduction

To elucidate the molecular mechanisms underlying hormone-related responses to FM treatment, we examined transcriptomic alterations in genes associated with the plant hormone signal transduction pathway (ko04075). Comparative analysis between FM1 and FM4 treatments revealed significant upregulation of several key genes involved in hormone signaling (Table S4), indicating that FM modulates hormonal responses in a concentration-dependent manner. For instance, *Zlat_10043500*, encoding a U-box domain-containing protein homologous to Arabidopsis *PUB16*, exhibited a substantial increase in expression from an average FPKM of 5.74 in FM1 to 34.62 in FM4. This gene is implicated in brassinosteroid-mediated signaling and abiotic stress responses. Similarly, *Zlat_10008675*, annotated as a chitin-inducible gibberellin-responsive protein (*CIGR2* homolog), showed a marked rise in expression (43.77 to 125.90 FPKM), suggesting enhanced gibberellin signaling under higher FM exposure. In addition, *Zlat_10018163* and *Zlat_10019537*, which correspond to *PUB27* and *PUB21* respectively—both regulators of hormone signaling via the ubiquitin-proteasome pathway—were also significantly induced, indicating enhanced post-translational regulation of signaling components. Notably, *Zlat_10026590*, annotated as a TIFY 11b protein and homologous to Oryza *JAZ* repressors, was dramatically upregulated from 20.13 to 134.34 FPKM, suggesting suppression of jasmonic acid signaling. The overall pattern suggests that FM treatment suppress multiple hormone signaling pathways, particularly jasmonate and brassinosteroid signaling (Figure S6).

In addition to the FM1 vs. FM4 comparison, analysis of differential gene expression in CK vs. FM1 and CK vs. FM4 treatments further confirmed the perturbation of hormone signaling pathways by fenaminosulf (Tables S2 and S3). In the CK vs. FM4 group, *Zlat_10016729*, annotated as a probable indole-3-acetic acid-amido synthetase (*GH3* family), was significantly upregulated under FM exposure, with FPKM values increasing from below 1.0 in CK to over 5.0 in FM4. This enzyme plays a crucial role in auxin homeostasis by conjugating free indole-3-acetic acid (IAA), thereby attenuating auxin signaling. Similarly, *Zlat_10018163*, a *PUB27* homolog already identified in the FM1 vs. FM4 comparison, was consistently upregulated from 8.45 FPKM in CK to over 20 FPKM in FM4, reinforcing its role in ubiquitin-mediated regulation of hormone responses. In contrast, *Zlat_10018131*, a homolog of auxin-responsive gene *IAA4*, was markedly downregulated in FM4 compared to CK, with expression decreasing from approximately 44.2 to 21.2 FPKM, suggesting suppression of early auxin signaling components.

Collectively, these expression profiles suggest that FM treatment disrupts multiple hormone signaling networks, particularly those associated with auxin, brassinosteroid, gibberellin, and jasmonate pathways. These changes collectively suggest a complex and concentration-dependent reprogramming of hormone signaling under FM treatment, likely suppressing growth-promoting and defense-related responses. This multi-layered mis-regulation of hormonal homeostasis led to conditions more conducive to pathogen invasion and gall formation in *Z. latifolia*.

## 3. Discussion

The current study provides a comprehensive investigation into the multifaceted effects of Fenaminosulf (FM) on *Z. latifolia*, revealing a complex interplay between chemical treatment, plant physiology, and molecular responses. The findings demonstrate that FM exerts concentration-dependent impacts on plant growth, development, and molecular mechanisms, with profound implications for agricultural practices and understanding plant-chemical interactions.

The growth response to FM treatment exhibited a nuanced concentration-dependent pattern, where moderate concentrations (1.25 g/L, FM4) stimulated vegetative growth, while higher concentrations (5 g/L, FM1 and 2.5 g/L, FM2) either stagnated or inhibited plant development. This non-linear response highlights the critical importance of precise chemical application in agricultural settings. The observed variations in plant height, leaf length, and stem thickness suggest that FM can act as a growth regulator by modulating physiological processes to enhance productivity and resilience [23,24,25]. Chlorophyll content analysis further illuminated the physiological impacts of FM, with moderate concentrations maintaining or slightly increasing photosynthetic capacity, while higher concentrations significantly reduced chlorophyll levels. This reduction likely stems from oxidative stress induced by excessive chemical exposure, which can disrupt chloroplast function and impair light-harvesting efficiency [26,27]. While, under moderate FM treatment, growth promotion might be driven primarily by factors other than a significant increase in chlorophyll concentration, such as optimized hormonal balance or resource allocation, as supported by our transcriptomic data. The findings underscore the delicate balance between chemical stimulation and physiological stress in plant systems [28,29].

The most striking result was FM’s pronounced effect on gall formation, a critical agricultural trait for *Z. latifolia* production. High FM concentrations significantly enhanced gall development, with FM2 showing the most consistent effect. This promotion of gall formation appears to be mediated through complex molecular mechanisms involving hormonal regulation and plant-pathogen interactions. Transcriptomic analysis revealed molecular shifts underlying these physiological changes. The significant modulation of genes involved in phenylpropanoid biosynthesis, flavonoid metabolism, and plant-pathogen interactions provides insights into the molecular basis of FM’s effects. The suppression of the pattern recognition receptor kinase *Xa21* [30,31,32,33] and the downstream signaling kinase *PBL19* [33,34,35], both critical for PAMP-triggered immunity, along with the inhibition of defense-related hormone pathways like jasmonic acid and brassinosteroid signaling, strongly suggests that high FM concentrations compromise the plant’s ability to effectively recognize and mount a defense against *Ustilago esculenta*. This weakened defense state likely creates a more permissive environment for the fungus to establish the intimate interaction required to induce gall formation.

Hormone signaling analysis uncovered another critical mechanism of FM’s action. The inhibition of auxin, cytokinin, brassinosteroid, and jasmonic acid metabolism suggests a fundamental reprogramming of plant developmental and defense strategies [4,36,37]. By disrupting these hormonal pathways, FM appears to redirect plant resources and physiological responses, potentially making *Z. latifolia* more susceptible to the symbiotic smut fungus *U. esculenta* crucial for gall formation. These results align with the previous findings [4,5,38].

The observed changes in MAPK signaling [39,40,41], ABC transporters [42,43], and glutathione metabolism [44,45] further demonstrate the comprehensive molecular recalibration induced by FM treatment. These pathways are integral to stress response and adaptation [39,40,41,42,43,44,45], indicating that FM triggers a sophisticated reprogramming of plant cellular mechanisms beyond simple growth promotion.

While the results offer promising insights into chemical-mediated plant manipulation, they also emphasize the need for precise and controlled application. The concentration-dependent responses highlight the potential risks of indiscriminate chemical use in agriculture. The fine line between growth stimulation and physiological stress necessitates careful, targeted approaches to chemical treatment.

The study opens numerous avenues for future research. Investigating the precise molecular interactions between FM, plant hormonal networks, and fungal colonization could provide deeper understanding of plant-chemical-pathogen relationships. Moreover, exploring genetic modifications or biotechnological approaches to manipulate key resistance-related genes could lead to more resilient and productive *Z. latifolia* cultivars.

## 4. Materials and Methods

### 4.1. Plant Materials and Growth Conditions

The experiment was conducted using the *Z. latifolia* cultivar “Meirenjiao”. Near-ground stems of *Z. latifolia* were sterilized and cultivated in a nutrient solution following the methodology of Chen et al., [15]. The plants were grown in plastic containers (60 cm × 40 cm × 35 cm) and maintained in a hydroponic incubator under controlled conditions for two weeks to obtain fresh seedlings. The incubation conditions were set at a temperature of 28 °C ± 2 °C, with a 14-h light and 10-h dark cycle and a relative humidity of 50–60%. When the seedlings reached a height of 8–10 cm, they were transferred to a greenhouse and grown for 30 days until reaching the seven-leaf stage. Plants were cultivated in an insect-free phytotron at the Lishui Institute of Agriculture and Forestry Sciences, where the environmental conditions were maintained at a day/night temperature of 28 ± 1 °C/24 ± 1 °C, relative humidity of 60 ± 5%, and a photoperiod of 16 h of light and 8 h of darkness.

### 4.2. Fenaminosulf (FM) Treatment and Sample Collection

To investigate the effects of Fenaminosulf (FM) treatment on *Z. latifolia*, 21 healthy plants were placed in each of twelve plastic containers and subjected to different FM treatment conditions. The 12 containers were divided into four groups with three replicate containers assigned per group: a control group (CK) that received no FM treatment and three experimental groups that received three applications of 50% FM WP (Yuelian Chemical Co., Ltd., Shanghai, China) at concentrations of 5 g/L (FM1), 2.5 g/L (FM2), and 1.25 g/L (FM4) at seven-day intervals. The treatment process was repeated three times, and all experimental units were provided with the same fertilizer, water management, and pest control practices.

Physiological measurements were recorded at 7, 14, and 21 days after foliar FM treatment, including plant height, leaf length, stem diameter, and chlorophyll content. Plant height was measured from the base of the plant to the top of the stem. Leaf length was measured for the third fully expanded leaf from the top, from the leaf sheath junction to the leaf tip. A digital caliper was used to record the stem diameter at the widest point perpendicular to the plant’s growth axis. The total chlorophyll content was measured on three fully opened leaves using a SPAD meter, with three technical replicates per leaf. After 70 to 80 days following FM application, the gall size was measured, and once 1–2 cm of the white flesh pseudo-stem was exposed, the plants were considered ripe for harvest. At this point, the gall formation rate was recorded. Seven days post-treatment, leaf samples (middle section of three leaves) were collected for plant hormone and transcriptomics analyses, immediately frozen in liquid nitrogen, and stored at −80 °C until further use.

### 4.3. Transcriptome Sequencing and RNA Extraction

To examine the molecular responses of *Z. latifolia* to FM treatment, RNA sequencing was performed. Leaf samples were collected from three biological replicates per treatment group, immediately frozen in liquid nitrogen, and stored at −80 °C before RNA extraction. Total RNA was extracted using the TRIzol reagent (Invitrogen, Carlsbad, CA, USA) following the manufacturer’s instructions. The RNA concentration and purity were determined using a NanoDrop 2000 spectrophotometer (Thermo Scientific, Waltham, MA, USA), and RNA integrity was confirmed by agarose gel electrophoresis (1% *w*/*v*) and an Agilent 2100 Bioanalyzer (Agilent Technologies, Santa Clara, CA, USA).

### 4.4. RNA Library Preparation and Illumina Sequencing

For transcriptome sequencing, mRNA was enriched using oligo(dT) beads to capture polyadenylated transcripts, fragmented into short sequences (200–300 bp), and used as a template for first-strand cDNA synthesis with random hexamer primers and reverse transcriptase. Second-strand cDNA synthesis was performed using DNA polymerase I and RNase H. The cDNA fragments were purified using AMPure XP beads (Beckman Coulter, Brea, CA, USA), and adapters were ligated to the purified cDNA. The libraries were amplified through PCR, and the final quality of the cDNA libraries was assessed using an Agilent 2100 Bioanalyzer. The cDNA libraries were sequenced on the Illumina NovaSeq 6000 platform (Illumina, San Diego, CA, USA), generating paired-end reads of 150 bp. A sequencing depth of at least 6 Gb per sample was ensured to provide sufficient transcriptome coverage.

Raw sequencing reads were evaluated for quality using FastQC (v0.11.9), and low-quality reads and adapters were removed using Trimmomatic (v0.39). High-quality clean reads were then mapped to the *Z. latifolia* reference genome (if available) using HISAT2 (v2.2.1). If a reference genome was not available, de novo transcriptome assembly was performed using Trinity (v2.13.2).

### 4.5. Differential Gene Expression Analysis

Gene expression levels were quantified using FeatureCounts (v2.0.3) and reported in fragments per kilobase of transcript per million mapped reads (FPKM). The gene count matrices were normalized using the Trimmed Mean of M-values (TMM) method. Differentially expressed genes (DEGs) were identified using DESeq2 (v1.34.0) in R, applying a log2 fold change (FC) threshold of ≥|1.5| and an adjusted *p*-value (false discovery rate, FDR) < 0.05 for statistical significance. Functional annotation of DEGs was performed using the NR (NCBI non-redundant), Swiss-Prot, Pfam, and KEGG databases through BLASTX (E-value < 1 × 10^−5^). Gene Ontology (GO) enrichment analysis was conducted using GOseq (v1.50.0) to classify DEGs into biological processes, molecular functions, and cellular components. KEGG pathway enrichment analysis was performed using KOBAS (v3.0) to identify significantly enriched metabolic pathways, with pathways showing an FDR < 0.05 considered significantly enriched.

### 4.6. Statistical Analysis

All statistical analyses were conducted using GraphPad Prism 9.0 and R 4.2.0. Differences among treatment groups were evaluated using one-way ANOVA followed by Tukey’s HSD test for post hoc multiple comparisons. Statistical significance was set at *p* < 0.05. Data are presented as means ± standard error (SE).

## 5. Conclusions

This research provides a comprehensive molecular view of how Fenaminosulf modulates *Z. latifolia* growth and development. By revealing the intricate mechanisms underlying chemical-induced physiological changes, the study contributes significantly to our understanding of plant responses to chemical treatments and offers valuable insights for agricultural innovation and crop management strategies.

## Data Availability

The datasets generated and/or analyzed during the current study are available at NCBI; Transcriptome sequencing data (PRJNA1248149).

## Supplementary Materials

The following supporting information can be downloaded at: https://www.mdpi.com/article/10.3390/plants14111628/s1, Figure S1. Heatmaps illustrating expression profiles in three pairwise comparisons (**A**) CK vs. FM1, (**B**) CK vs. FM4, and (**C**) FM1 vs. FM4; Figure S2. KEGG pathway enrichment for three pairwise comparisons; Figure S3. Expression dynamics of DEGs identified in FM-Treated *Z. latifolia* in comparison FM1 vs FM4; Figure S4. Phenylpropanoid pathway dynamics under (**A**) CK vs. FM1 and (**B**) CK vs. FM4 comparisons; Figure S5. Plant-pathogen interaction pathway dynamics under (**A**) CK vs. FM1 and (**B**) CK vs. FM4 comparisons; Figure S6. Plant hormone signal transduction pathway dynamics under (**A**) CK vs. FM1 and (**B**) CK vs. FM4 comparisons; Table S1. Summary statistics and quality control of the transcriptomic data; Table S2. Differential regulation and annotation information of DEGs identified in CK vs. FM1 comparison; Table S3. Differential regulation and annotation information of DEGs identified in CK vs. FM4 comparison; Table S4. Differential regulation and annotation information of DEGs identified in FM1 vs. FM4 comparison.
