# Supplementary figures and images for: Fenaminosulf Promotes Growth and Gall Formation in Zizania latifolia Through Modulation of Physiological and Molecular Pathways
Source: Plants (Basel). 2025 May 27;14(11):1628. doi: 10.3390/plants14111628 (PMC12157912; doi:10.3390/plants14111628)

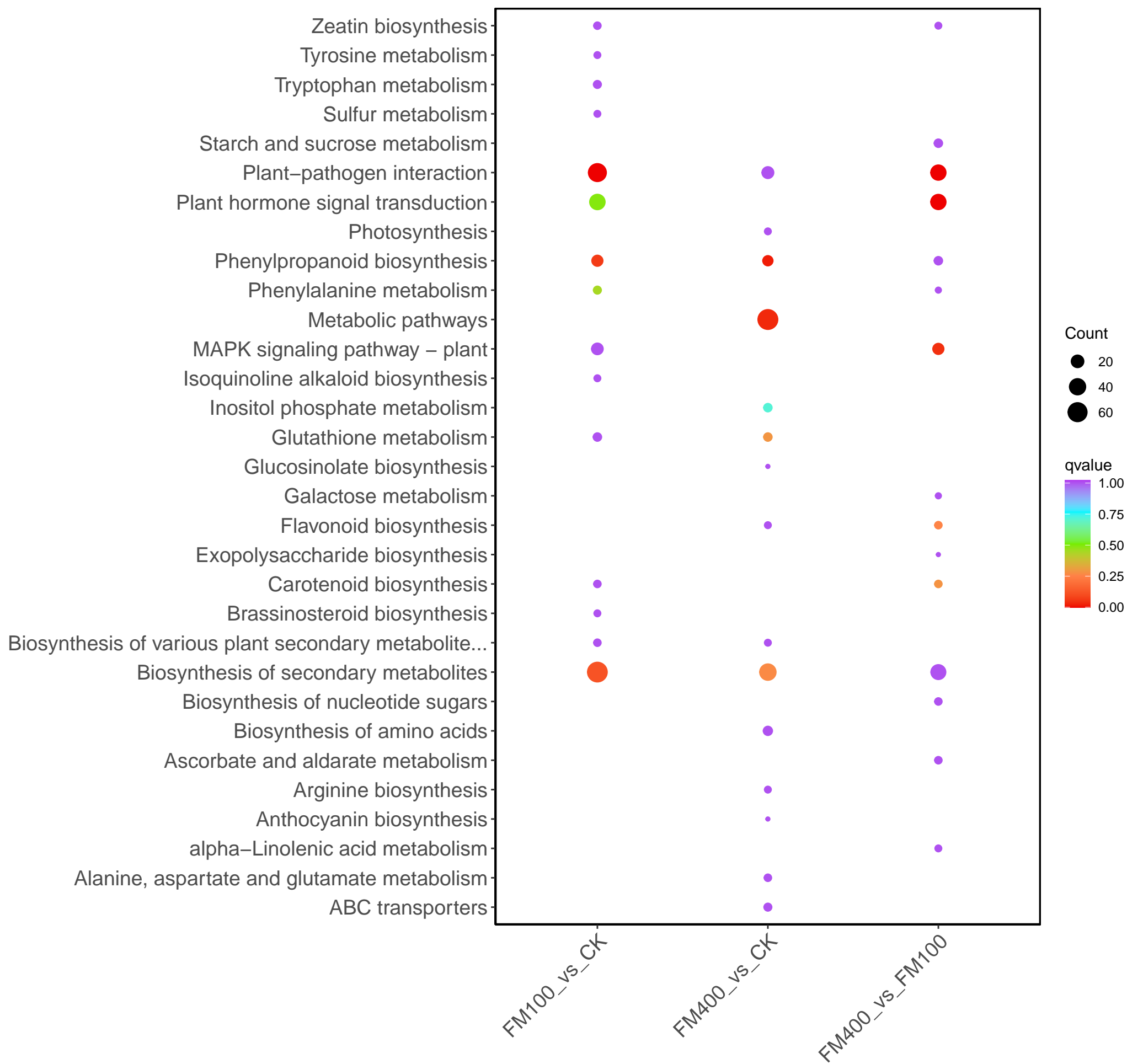

Supplement: Supplementary file 1 [file plants-14-01628-s001.zip › Figure S2.pdf]

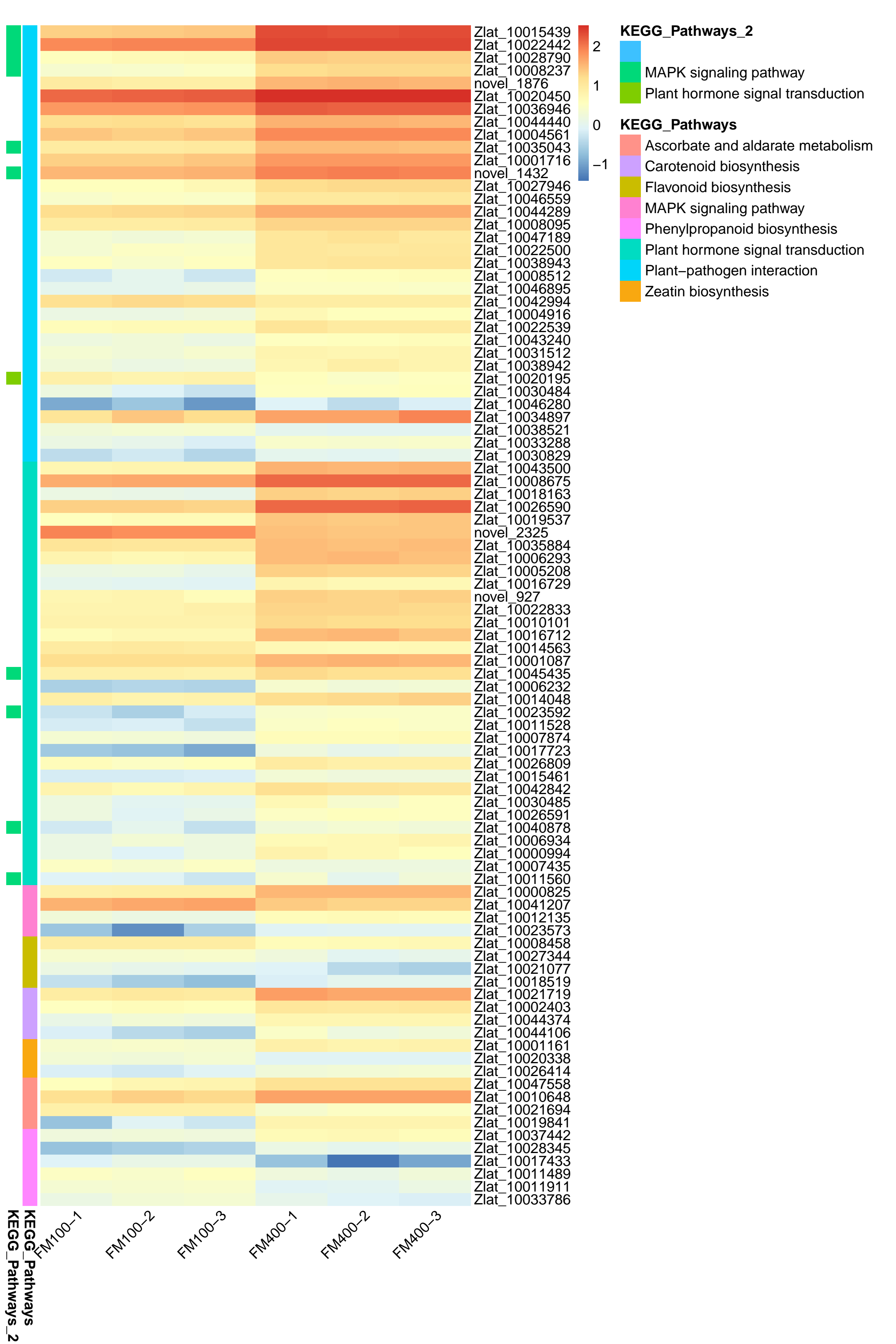

Supplement: Supplementary file 1 [file plants-14-01628-s001.zip › Figure S3.pdf]

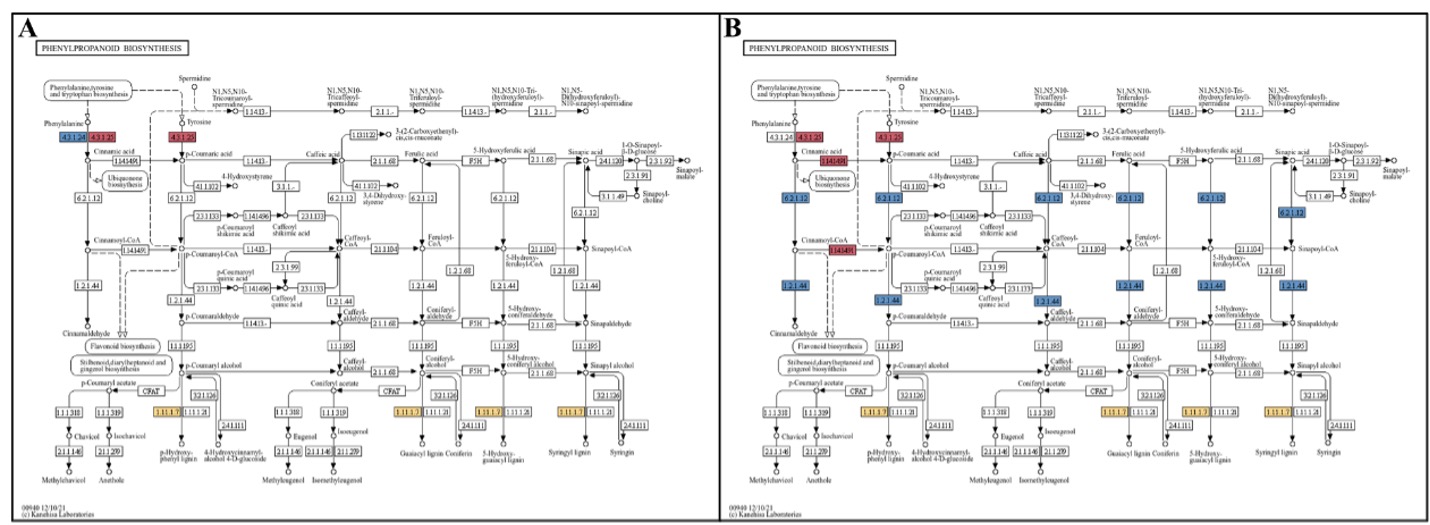

Supplement: Supplementary file 1 [file plants-14-01628-s001.zip › Figure S4.jpg]
